# “The Influence of Academic Training on Cognitive Reserve: A Study in Older Adults with Mild and Subjective Cognitive Impairment”

**DOI:** 10.1002/alz70858_102321

**Published:** 2025-12-24

**Authors:** Maria Belen Martin Escandell, Florencia Vallejos, Guido Santiago Dorman, Julian Fernandez Boccazzi, Ignacio Flores, Santiago O'Neill

**Affiliations:** ^1^ Neuroscience Institute, Favaloro Foundation, Buenos Aires, Buenos Aires, Argentina; ^2^ Neuroscience Institute, Favaloro Foundation, Buenos Aires, Argentina

## Abstract

**Background:**

Cognitive reserve allows the brain to withstand damage and aims to maintain proper functioning despite the presence of brain lesions or neurodegenerative diseases. However, it is not known whether there is a specific relationship between cognitive reserve and individual intellectual development throughout life, such as the stimulation associated with certain academic background. The main objective of this study is to determine if academic training influences the performance of specific cognitive domains in highly educated middle‐aged and older adults with subjective cognitive decline (SCD) and mild cognitive impairment (MCI).

**Method:**

This is a cross‐sectional observational study. Patients over 50 years with more than 15 years education level and diagnosed with subjective memory complaints and/or MCI that attended to a memory clinic were included. Patients were categorized according to the ISCO‐08 (International Standard Classification of Occupations 2008). All participants underwent cognitive evaluation, including memory, attention, language, executive functions and visuospatiality Differences between occupational categories and cognitive domains were evaluated. Statistical analysis was conducted using RStudio. Descriptive statistics and comparison of variances of means were performed using ANOVA. The contrasts between occupations were evaluated using Tukey's technique.

**Result:**

A total of 617 patients were recruited, with an average age of 67 years ± 9.1 years and 17 ± 2.4 years of schooling; 69.2% of the participants were women. Patients in business administration showed strength in executive functions over other occupations (*p* < 0.05), while those in teaching, science, engineering, and technology showed superior linguistic skills ( *p* < 0.05). Additionally, health professionals, legal experts, educators, and those in science, engineering, and technology demonstrated better results in memory tests compared to those in business administration.

**Conclusion:**

This study suggests that previous educational background could influence the cognitive performance in subjects with SCD and MCI showing that cognitive reserve can be selective. Educational background may provide strengths and weakness in specific cognitive domains. More studies should be performed to confirm these findings.

